# Preeclampsia: Insights into pathophysiological mechanisms and preventive strategies

**DOI:** 10.1016/j.ajpc.2025.101054

**Published:** 2025-07-03

**Authors:** Chiara Martini, Zeeba Saeed, Paola Simeone, Stefano Palma, Mirella Ricci, Allegra Arata, Anna Sorella, Rossella Liani, Fabrizio Ricci, Francesco D’Antonio, Anna Vittoria Mattioli, Sabina Gallina, Francesca Santilli, Giulia Renda

**Affiliations:** a Department of Neuroscience, Imaging and Clinical Sciences, G. d’Annunzio University of Chieti-Pescara, Chieti, Italy; b Center for Advanced Studies and Technology, G. d’Annunzio University of Chieti-Pescara, Chieti, Italy; c Department of Medicine and Aging, G. d’Annunzio University of Chieti-Pescara, Chieti, Italy; d University Cardiology Division, SS. Annunziata Hospital, Chieti, Italy; e Center for Fetal Care and High-risk Pregnancy, Department of Obstetrics and Gynecology, G. d’Annunzio University of Chieti-Pescara, Chieti, Italy; f Department of Quality of Life Sciences, University of Bologna – Alma Mater Studiorum, Bologna, Italy

**Keywords:** Abnormal placentation, Endothelial dysfunction, Hypertension, Low-dose aspirin, Preeclampsia, Pregnancy

## Abstract

Preeclampsia is a hypertensive disorder of pregnancy associated with significant maternal and fetal complications. Its pathogenesis involves endothelial dysfunction, abnormal placentation, and coagulation abnormalities, leading to increased thrombotic and hemorrhagic risks. This narrative review provides an in-depth overview of the pathophysiological mechanisms underlying preeclampsia, with a particular focus on its thrombotic and hemorrhagic complications. Treatment strategies are explored, with emphasis on the role of low-dose aspirin in reducing early-onset preeclampsia. However, aspirin’s effectiveness varies based on dosage and timing, with higher doses showing greater benefit in preventing severe preeclampsia. Despite aspirin’s widespread use, further optimization of its therapeutic role remains necessary to enhance maternal and fetal outcomes.

## List of abbreviations

APTT: Activated partial thromboplastin time
CVD: Cardiovascular diseases
FGR: Fetal growth restriction
IUGR: Intrauterine growth restriction
LDA: Low-dose aspirin
LMWH: Low-molecular-weight heparin
MPs: Microparticles
NO: Nitric oxide
PlGF: Placental growth factor
PT: Prothrombin time
RAS: renin–angiotensin system
sEng: soluble endoglin
sFlt-1: soluble fms-like tyrosine kinase-1
TFPI: Tissue factor pathway inhibitor
T-PA: Tissue-type plasminogen activator
TXA_2_: ThromboxaneA_2_
VEGF: Vascular endothelial growth factor
VTE: Venous thromboembolism

## 1 Introduction

Preeclampsia is a complex multisystem disease, defined as new-onset hypertension, which usually occurs in a pre-gestational normotensive patient, typically after 20 weeks of gestation, and evidence of end-organ dysfunction in the presence or absence of proteinuria [1,2]. Preeclampsia commonly impacts 2 %−8 % of pregnancies, leading to substantial maternal and perinatal morbidity and mortality, especially in early-onset cases. Worldwide, approximately 76,000 women and 500,000 infants annually suffer from this condition. The economic impact includes increased cesarean deliveries, preterm births, and significant adverse outcomes, imposing a substantial burden on patients and healthcare systems [3].

**Figure:**
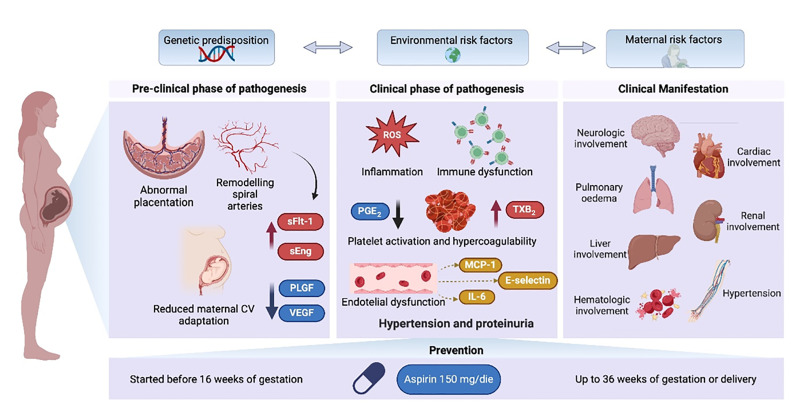
**Central Illustration.** Pathogenesis of preeclampsia and clinical manifestations Genetic, environmental, and maternal risk factors lead to abnormal placentation, reduced maternal cardiovascular adaptation, and insufficient remodeling of spiral arteries. The release of antiangiogenic markers such as soluble fms-like tyrosine kinase-1 (sFlt-1) and soluble endoglin (sEng) in maternal circulation results in inflammation, immune dysfunction, hypercoagulability, platelet and endothelial dysfunction exerting a negative impact on maternal and fetus organ systems and causing hematological complications. For high-risk populations, low-dose aspirin treatment is recommended. IL-6, interleukin 6; MCP-1, monocyte chemotactic protein-1; PGE2, prostaglandin E2; PLGF, placental growth factor; sEng, soluble endoglin; sFlt-1, soluble fms-like tyrosine kinase-1; TXB_2_, thromboxaneB_2_; VEGF, vascular endothelial growth factor. (Created with Biorender.com).

The pathogenesis of preeclampsia is multifactorial, caused by genetic, environmental, and maternal risk factors leading to abnormal placentation, reduced maternal cardiovascular adaptation, and insufficient remodeling of spiral arteries. The abnormal placentation increases the release of antiangiogenic markers in maternal circulation, resulting in inflammation, immune dysfunction, hypercoagulability, platelet, and endothelial dysfunction. This exerts a negative impact on maternal and fetus organ systems and hematological complications (**Central Illustration**). Low-dose aspirin (LDA) is an effective treatment for reducing the risk of early-onset preeclampsia [1,4,5] in high-risk women by decreasing platelet activation and inflammation [6]. Given that efficacy varies due to factors such as dose and gestation [7,8], personalized management is crucial [7].

This narrative review provides an in-depth overview of the pathophysiological mechanisms underlying preeclampsia, with a particular focus on its thrombotic and hemorrhagic complications. Treatment strategies are explored, with emphasis on the role of LDA in reducing early-onset preeclampsia.

## 2 Methods

The authors conducted the literature research between November 2023 and March 2025, using PubMed, ScienceDirect and Google Scholar as the primary sources. The search strategy focused on identifying the studies that particularly addressed the following outcomes in patients with preeclampsia: (a) the coagulation profile of preeclampsia patients, with an emphasis on endothelial and platelet dysfunction, coagulopathies, and microparticle production; (b) the clinical presentation and diagnostic features of preeclampsia; (c) bleeding and thrombotic complications; and (d) therapeutic strategies of preeclampsia involving aspirin, low-molecular-weight heparin (LMWH), and statins. Recent and high-impact studies were prioritized. The results were presented using a narrative approach due to the wide heterogeneity in study designs, populations, and outcomes across the available literature, which precluded a formal systematic synthesis. Studies were selected based on expert judgment of their relevance and scientific contribution, rather than through structured inclusion/exclusion criteria. The primary search terms used were ‘cardiovascular complication’, ‘hypercoagulation or coagulopathies’, ‘microparticles’ or ‘extracellular vesicles’, ‘clinical presentation’, ‘thrombotic complication’, ‘hemorrhage risk’, ‘aspirin’, ‘low molecular weight heparin’, and ‘statin,’ in combination with the term ‘preeclampsia’. Both original research articles and selected high-quality reviews were considered.

## 3 Classification and risk factors of preeclampsia

Preeclampsia is categorized into early-onset (<34 weeks) and late-onset (≥34 weeks). Additionally, it is subcategorized as preterm (delivery at <37 weeks) and term (delivery at ≥37 weeks). Early-onset preeclampsia carries a greater risk of adverse maternal and perinatal outcomes compared to late-onset or term preeclampsia [3].

Extensive research has examined risk factors associated with preeclampsia. Major factors include prior preeclampsia, chronic hypertension, pre-existing diabetes, antiphospholipid syndrome, and obesity. Additional risks comprise advanced maternal age, nulliparity, chronic kidney disease history, and assisted reproductive technology use [9,10]. These maternal conditions have been reported to be associated with endothelial dysfunction, which may contribute to increased risk of preeclampsia [11]. Genetic predisposition has been extensively investigated. African-American women have a higher risk than white women. Offspring of mothers with preeclampsia have increased risk. A genome-wide study linked a fetal genome single-nucleotide polymorphism near FLT1 to preeclampsia. Increased levels of soluble fms-like tyrosine kinase-1 (sFlt1) have been detected in instances of trisomy 13 and multifetal and molar pregnancies, indicating a heightened susceptibility to preeclampsia [11]. Tyrmi et al. [12]. identified 13 new loci significantly linked to preeclampsia and related maternal hypertension. These loci impact endothelial function, placental development, and immunity, suggesting shared genetic mechanisms between preeclampsia and cardiovascular diseases (CVD), highlighting pregnancy as a window to future cardiovascular health.

Recent studies point to hyperlipidemia as a risk factor for preeclampsia. Lipid metabolism undergoes significant physiological changes during normal pregnancy. From the second trimester onward, levels of total cholesterol, LDL-C, HDL-C, triglycerides, and non-HDL cholesterol increase markedly [13]. Total cholesterol and LDL-C can rise by up to 50–60 %, and triglycerides may increase by as much as 200 %, with values peaking in the third trimester [14]. These changes support placental hormone production, maternal fat storage, and fetal membrane development. Dyslipidemia in pregnancy is defined by lipid levels above the 95th percentile (for total cholesterol, triglycerides, LDL-C) or below the 5th percentile (for HDL-C) for gestational age. Severe hypertriglyceridemia (>1000 mg/dL) is rare and usually occurs in late pregnancy [15].

A large European cohort study found that high triglycerides levels in the first trimester, but not total cholesterol, were independently associated with adverse outcomes, such as gestational hypertension, preeclampsia, and large-for-gestational-age preterm births [16].

Maternal hypercholesterolemia has been proposed as a potential risk modifier and warrants further study [17,18]. Current practice does not routinely include lipid testing pre-conception or during pregnancy. Professional, societal recommendations should advocate for hyperlipidemia screening, followed by appropriate management, pre-conception and during pregnancy [19].

Diet has become a focus of growing concern as a risk factor in the etiology of preeclampsia. Several studies have reported that micronutrient deficiency is associated with increased risk of developing this disorder. A meta-analysis [20] of 27 randomized clinical trials reported that vitamin D supplementation significantly reduced the risk of preeclampsia, especially when administered before 20 weeks of gestation. Adequate calcium intake has also been shown to help prevent preeclampsia during pregnancy. Meertens et al. [21]. focused on calcium supplementation and recommended that it can lead to significant reductions seen in the incidence of this condition. A prospective cohort study [22] also reported that multivitamin/mineral supplementation in the first-trimester was associated with a reduced risk of preeclampsia, particularly among obese and overweight women, indicating that micronutrient support in the early weeks of pregnancy may modulate the inflammatory pathway or oxidative stress-induced mechanisms involved in preeclampsia pathogenesis.

## 4 Pathophysiology of preeclampsia

**Fig. 1:**
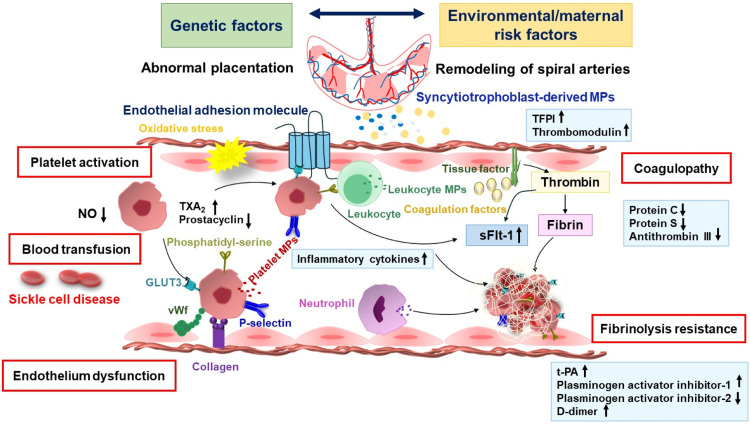
Pathophysiology of preeclampsia A complex combination of genetic and environmental/maternal risk factors are involved in the development of preeclampsia. Circulating syncytiotrophoblast-derived MPs reveal placental injury and trigger hypercoagulability. Preeclamptic women exhibit high levels of MPs derived from platelets, endothelial cell, leukocytes, and monocytes. Endothelial dysfunction leads to platelet aggregation and adhesion. Elevated levels of activated platelets enhance platelet-leukocyte aggregation, resulting in a inflammatory and prothrombotic state. Transfusing erythrocytes promote thrombosis in preeclamptic women. GLUT3, glucose transporter 3; MPs, microparticles; NO, nitric oxide; sFlt-1, soluble fms-like tyrosine kinase-1; TFPI, tissue factor pathway inhibitor; t-PA, Tissue-type plasminogen activator; TXA_2_, thromboxaneA2; vWf, von Willebrand factor.

The pathogenesis of preeclampsia is complex and remains unclear. A multifactorial combination of genetic and environmental/maternal risk factors, along with abnormal placentation, reduced maternal cardiovascular adaptation, insufficient remodeling of spiral arteries, inflammation, immune dysfunction, hypercoagulability, platelet dysfunction, and endothelial dysfunction, is suggested to be involved in the pathophysiology of preeclampsia [9,10] (Fig. 1).

### 4.1 Abnormal placentation

It is believed that preeclampsia advances in two stages: a preclinical phase characterized by abnormal placental development with incomplete spiral artery remodeling in the early first trimester and a clinical phase in the later second and third trimesters, characterized by an excess of antiangiogenic factors, leading to a “maternal syndrome” featuring hypertension, proteinuria, and organ dysfunction due to placental hypoxia [23]. During normal implantation, placental trophoblasts migrate into the maternal uterine spiral arteries, prompting extensive remodeling that eliminates their smooth muscle layer. This invasion extends deeply into the spiral artery, resulting in extensive remodeling of maternal spiral arterioles into high-capacity and high-flow vessels, facilitating increased blood flow to nourish the fetus independently of maternal vascular adjustments. However, in preeclampsia, cytotrophoblasts fail to undergo the required transformation from a proliferative epithelial subtype to an invasive endothelial subtype, resulting in incomplete artery remodeling. This deficiency leads to narrowing of maternal vessels and relative placental ischemia [24]. Placental ischemia prompts the release of antiangiogenic factors such as sFlt-1 and soluble endoglin (sEng) into the maternal circulation. These factors antagonize proangiogenic molecules like vascular endothelial growth factor (VEGF) and placental growth factor (PlGF), impairing angiogenesis. This imbalance contributes to maternal hypertension, proteinuria, and organ dysfunction. Understanding these mechanisms highlights the importance of early detection to prevent adverse outcomes [25].

### 4.2 Endothelial dysfunction

Endothelial integrity is vital for a successful pregnancy. In physiological conditions, the endothelium exhibits a negatively charged, non-adhesive glycosaminoglycan layer that impedes thrombin generation and the adhesion of platelets and leukocytes. The intact endothelial layer expresses various anticoagulant proteins, including thrombomodulin, the endothelial protein C receptor, and tissue plasminogen activator [26]. Abnormal placentation leading to endothelial dysfunction is key to pathophysiology of preeclampsia, and it occurs before the clinical manifestation of preeclampsia. In preeclampsia, placental ischemia induces systemic endothelial damage through the release of antiangiogenic factors and inflammatory mediators, disrupting vascular homeostasis [25]. Endothelial adhesion molecules on the endothelium of maternal and the uterine placental bed facilitate neutrophil and platelet recruitment [26], while increased expression of molecules like intercellular adhesion molecule 1 enhances inflammatory cell adhesion and endothelial derived microparticles (MPs) release [26]. In preeclampsia pathophysiology, oxidative stress reduces nitric oxide (NO) availability via endothelial NO synthase inhibition or NO reaction with superoxide to form peroxynitrite [27]. NO mediates vasorelaxation, anti-aggregation, and is crucial in placentation and VEGF production, besides inhibiting leukocyte adhesion and platelet aggregation. Reduced NO availability contributes to abnormal placentation; these changes heighten vascular resistance and promote hypertension [27]. Elevated levels of von Willebrand factor, endothelin, thrombomodulins, and fibronectin in maternal circulation activate endothelial cells [27], leading to platelet aggregation and adhesion with consequent increase in abnormal coagulation due to collagen exposure [25]. Increased thromboxaneA2 (TXA_2_) and decreased prostacyclin production disrupt the prostacyclin/TXA_2_ ratio, reducing uteroplacental blood flow and exacerbating placental ischemia and endothelial damage [27]. Endothelial dysfunction and damage in preeclampsia can compromise the anticoagulant activity of activated protein C and increase exposure to sub-endothelial tissue factor, triggering blood coagulation [26].

### 4.3 Platelet dysfunction

During late first-trimester pregnancy, uterine arteries undergo remodeling as extravillous trophoblasts invade, replacing vascular smooth muscle and endothelium and creating vessels which facilitate placental perfusion. The perturbation of this process leads to reduced perfusion, placental stress, and platelet accumulation in the partially disrupted placenta [24,28]. Platelet activation play a key role in the pathophysiology of preeclampsia, by causing hemostatic abnormalities [29]. Procoagulant activity of platelets may cause impaired placental circulation, potentially causing fetal growth restriction (FGR) [30]. Nowaczyk et al. [30]. found that in pregnancies complicated by FGR, maternal platelets exhibit elevated reactive oxygen species and defective metabolism, so platelet dysfunction, rather than count or volume, correlates with FGR. Moreover, platelet redox imbalance contributes to oxidative damage and modulates platelet activation, impacting NO bioavailability. Although challenging to measure, intraplatelet reactive oxygen species warrant examination for potential interventions in preventing FGR. Targeting platelet mitochondria, crucial in generating reactive oxygen species, offers a promising therapeutic strategy to mitigate impaired oxygen metabolism and platelet dysfunction in preeclampsia. Thrombocytopenia, the most common hemostatic abnormality in pregnancy-induced hypertension, worsens with disease severity and is caused by platelet clearance and hemolysis in preeclampsia. In preeclampsia, platelet adhesion to endothelial cells induces inflammatory cytokine release, causing severe hemostatic, vascular, and coagulation abnormalities, leading to serious feto-maternal complications [29]. Elevated levels of activated platelets enhance platelet-leukocyte aggregation, promoting a prothrombotic state. The resultant platelets-leukocyte aggregates upregulate sFlt-1 expression, exacerbating endothelial dysfunction and inflammation [31].

## 5 Mother and infants’ outcomes

Preeclampsia is linked to an elevated risk of short- and long-term cardiovascular and chronic diseases for both mothers and infants [2]. If preeclampsia is not treated, women are at risk of immediate life-threatening complications like acute kidney disfunction, hepatic failure and rupture, pulmonary oedema, eclampsia, brain injury, hematologic disturbance and even death [2,32]. In addition, women are at a higher risk of developing CVD, stroke, metabolic syndrome, cognitive impairment, and chronic end-stage renal disease later in life. Preeclampsia also correlates with FGR. Babies born to mothers with preeclampsia face potential complications in the medium and long term, including neurodevelopmental issues, insulin resistance, diabetes mellitus, coronary heart disease, and hypertension [2,32].

## 6 Clinical manifestation of pre-eclampsia

**Fig. 2:**
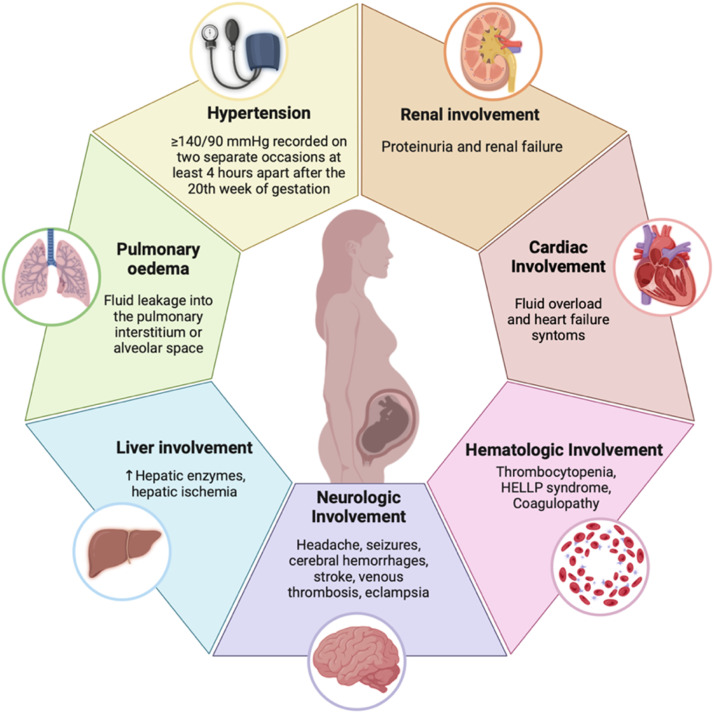
Clinical manifestation of preeclampsia The figure illustrates the systemic manifestations of preeclampsia, including hypertension, renal dysfunction with proteinuria, hepatic impairment, neurologic complications such as eclampsia, hematologic abnormalities leading to thrombotic complications, and cardiovascular involvement. These manifestations highlight the multifactorial impact of preeclampsia on maternal health and its potential complications. (Created with Biorender.com).

The clinical manifestations extend beyond elevated blood pressure, involving various organ systems including renal, hepatic, neurological, cardiac, hematologic, and pulmonary systems. Understanding these systemic effects is critical for timely diagnosis and management [2] (Fig. 2).

### 6.1 Hypertension

Gestational hypertension is defined by a systolic blood pressure equal to or exceeding 140 mmHg, and/or a diastolic blood pressure equal to or exceeding 90 mmHg, recorded on two separate occasions at least 4 h apart after the 20th week of gestation in a woman who previously had normal blood pressure. In cases of gestational hypertension with severe range blood pressures (a systolic reading of 160 mmHg or higher, and/or a diastolic reading of 110 mmHg or higher), a diagnosis of preeclampsia with severe features is warranted [1]. In preeclampsia, hypertension arises alongside increased systemic vascular resistance and afterload, involving the participation of the renin–angiotensin system (RAS) and immune mechanisms. While the RAS is typically upregulated in normal pregnancy, preeclampsia displays lower levels of renin, angiotensin II, and aldosterone. In normal pregnancies, AT1-receptor is down-regulated, whereas in preeclampsia, it enhances the RAS cascade by forming a heterodimer with bradykinin receptor B2 [33]. Furthermore, placental hypoxia triggers the production of stimulatory autoantibodies against the second extracellular AT1 receptor loop, intensifying vasoconstriction. These autoantibodies may contribute to angiotensin-II–induced vascular lesions in these patients, increasing sensitivity to angiotensin-II through elevated endothelin-1, and enhancing placental production of sFlt-1 and sEng [34].

### 6.2 Renal involvement

Proteinuria in preeclampsia results from increased renal tubular permeability to large-molecular-weight proteins and it is defined as 300 mg or more per 24-hour urine collection or protein/creatinine ratio of 0.3 or more or dipstick reading of 2+[1]. In physiological conditions, VEGF plays a crucial role in maintaining renal glomerular integrity by inducing the formation of endothelial fenestrations. Elevated sFlt-1 released from the hypoxic placenta and reduced NO contribute to renal tubular injury. High sFlt-1 levels inhibit podocyte-specific VEGF, leading to glomerular endothelial damage, termed glomerular endotheliosis, characterized by endothelial swelling and capillary narrowing. This disrupts glomerular filtration barrier and induces inter-podocyte separation, thereby fostering proteinuria. This, combined with reduced VEGF and PlGF availability, triggers endothelin 1 expression, promoting podocyte detachment from the glomerular endothelium [35]. Additionally, preeclampsia induces thrombotic microangiopathy in the kidneys due to hematologic abnormalities, increased proinflammatory cytokines, and reduced prostaglandin and NO levels, causing renal dysfunction [36]. In preeclampsia, renal insufficiency is characterized by serum creatinine levels exceeding 1.1 mg/dL or a twofold increase in serum creatinine concentration without any underlying renal pathology [1]. While glomerular complications often improve post-delivery, evidence suggests that preeclampsia elevates the risk of future chronic kidney diseases [36].

### 6.3 Liver involvement

Liver dysfunction in severe preeclampsia manifests with elevated alanine aminotransferase and aspartate aminotransferase levels, with alanine aminotransferase dominance indicating periportal necrosis. LDH elevation suggests hepatic ischemia and hemolysis, while coagulation parameter abnormalities reflect advanced disease. Alanine aminotransferase elevation surpassing aspartate aminotransferase initially aids in distinguishing preeclampsia from other liver diseases [1]. Liver dysfunction correlates with endothelial dysfunction, exacerbating hepatic microcirculatory compromise and hepatocellular necrosis. Elevated sFlt-1, inhibiting VEGF, alongside decreased NO synthase due to preeclampsia, synergistically induce liver dysfunction and thrombocytopenia. Liver rupture is a rare but life-threatening complication, associated with high maternal and perinatal mortality rates. Clinical presentation includes epigastric pain, shock, and collapse, with abdominal pain as a key initial symptom [37].

### 6.4 Neurological involvement

Preeclampsia presents significant neurological complications such as seizures, cerebral hemorrhages, stroke, venous thrombosis, posterior reversible encephalopathy syndrome, and eclampsia, driven by complex mechanisms. These events, occurring during and after pregnancy, are often unpredictable and can lead to maternal mortality if not promptly managed [38]. In preeclampsia, posterior reversible encephalopathy syndrome can manifest, presenting with symptoms like headache, seizures, visual disturbances, and other focal neurological deficits. Two main theories explain its pathophysiology: elevated blood pressure exceeding brain autoregulatory limits, causing cerebral hypertension and vasogenic edema, and endothelial dysfunction [37,38]. Eclampsia, characterized by seizures in pregnancy-induced hypertension, involves complex mechanisms, which may involve endothelial injury mitigation. Eclampsia's pathogenesis includes vasogenic and cytotoxic edema, possibly linked to cerebral blood flow dysregulation. Understanding these mechanisms aids in diagnosis and treatment, potentially preventing irreversible neurological damage [39].

### 6.5 Cardiac involvement

During a normal pregnancy, the cardiovascular system adjusts to support fetal development, with decreased peripheral resistance and a 30–50 % increase in cardiac output due to elevated heart rate and plasma volume. These changes prompt transient left ventricular eccentric hypertrophy, which resolves after childbirth. In preeclampsia, elevated vascular resistance due to impaired placentation exacerbates left ventricular remodeling, resulting in mild-to-moderate isolated left ventricular diastolic dysfunction and concentric hypertrophy. Unlike eccentric remodeling, which is a natural response, concentric remodeling reflects an abnormal cardiac adaptation to increased vascular resistance [37]. Preeclampsia increases the risk of peripartum cardiomyopathy, characterized by reduced left ventricular ejection fraction (<45 %) late in pregnancy or in early postpartum. Angiogenic imbalance and endothelial damage are key factors in both conditions, suggesting a possible causal relationship between preeclampsia and peripartum cardiomyopathy [40]. Preeclampsia increases the risk of future CVD, including venous thromboembolic events, stroke, myocardial infarction, and coronary heart disease [41,42]. Shared risk factors between preeclampsia and CVD include obesity, insulin resistance, metabolic abnormalities, dyslipidemia, elevated inflammatory responses, endothelial dysfunction, and hypercoagulable conditions. Persistent vascular damage and metabolic abnormalities may contribute to future cardiovascular complications in women with a history of preeclampsia [41]. Early detection of risk factors during pregnancy can mitigate adverse outcomes and predict future CVD. Understanding defective uteroplacental vascular remodeling is crucial to uncover the mechanisms linking preeclampsia to CVD [42]. Elevated inflammatory biomarkers like IL-6 and TNF-α correlate with CVD risk in preeclampsia. Reducing inflammation may improve endothelial function and lower CVD risk via NO-mediated mechanisms. This approach warrants exploration for reducing CVD risk in this populations [43].

### 6.6 Pulmonary oedema

Pulmonary oedema is a rare but severe complication of preeclampsia, contributing to maternal mortality. Multiple factors contribute to its development in pregnancy, including the use of tocolytic agents, cardiac disease, and fluid overload. Preeclampsia poses an elevated risk due to endothelial damage and reduced colloid osmotic pressure, resulting in fluid leakage into the pulmonary interstitium or alveolar space. When coupled with left ventricular dysfunction and increased peripheral vascular resistance, pulmonary oedema may ensue. Prompt recognition and management are essential to prevent adverse outcomes [44].

### 6.7 Hematologic involvement

#### 6.7.1 Mechanisms of thrombosis in preeclampsia

In late pregnancies, pregnant women experience a protective hypercoagulable state to prevent excessive bleeding during delivery. This state intensifies in placenta-related complications, like preeclampsia, leading to pathological prothrombotic state [31]. Altered hemostasis involves heightened platelet activation, fibrin accumulation, impaired fibrinolysis, and consumptive thrombocytopenia. Narrowed spiral arteries cause turbulent blood flow, activating coagulation cascades in the intervillous space [27,31]. Preeclamptic patients exhibit atherosclerotic lesions in spiral arteries, accompanied by activated platelets marked by TXA_2_, fibrin, complement deposits, and foam cells [27]. Soluble factors and extracellular vesicles released by placental lesions propagate, contributing to endothelial dysfunction and a hypercoagulable state in preeclamptic patients [31].

#### 6.7.2 Preeclampsia and venous thromboembolism

Thromboembolic complications can occur four to five times higher in healthy pregnant women compared to non-pregnant women due to the prothrombotic state of the blood, with the highest-risk phase being the postpartum period [26]. The underlying factors leading to venous thrombosis consist of hypercoagulability, venous stasis, and vascular damage of Virchow's triad, all persisting through pregnancy and extending into the postpartum period. High-risk factors for venous thromboembolism (VTE) include multiple birth, cesarean birth, preeclampsia, postpartum hemorrhage, placenta previa, advanced maternal age, low socioeconomic status, and premature rupture of membranes [45]. Particularly, preeclamptic patients are predisposed to a risk of VTE up to five times higher than the standard pregnancy-related risk documented in the general population, which can lead to death during pregnancy and the postpartum period [26]. Placenta previa correlates with antenatal thromboembolism, and postpartum hemorrhage is linked to postpartum thromboembolism. In both cases, blood transfusions are common [45]. In cases of placenta previa, frequent blood transfusions and the storage of red blood cells can increase the risk of thrombosis. Park et al. [45]. confirmed that preeclampsia is a postpartum, but not antenatal, risk factor for thromboembolism. This is attributed to vascular endothelial damage and the inherent thrombophilic disorder in preeclampsia, with limited delivery movement due to the mother's condition being another potential factor. Multiple deliveries cause more than a 2-fold increase in the risk of thromboembolism, as they are more associated with gestational hypertension, antenatal hemorrhage, and cesarean delivery. Cesarean section is related to a high risk of thrombosis due to elevated tissue trauma, the increased release of tissue factor, and long-term immobilization [45]. Establishing personalized risk prediction models for the development of preeclampsia and the associated risk of VTE is crucial to advance promising therapeutic approaches. Prioritizing research in this area has the potential to decrease morbidity and mortality in both mothers and neonates [26].

#### 6.7.3 Coagulopathy

Normal pregnancy exhibits a hypercoagulable state with increased coagulation and angiogenic factors, and decreased natural anticoagulant factors, like protein S, activated protein C and fibrinolysis, providing protection against bleeding during implantation and childbirth. In preeclampsia, this balance shifts dramatically, exacerbating hypercoagulability and leading to coagulopathy. Enhanced thrombin generation triggers fibrin formation and platelet activation, promoting hypercoagulation and impairing vascular function [25,31]. During placentation, tissue factor and plasminogen activator inhibitor 1 are the key modulators of hemostasis [46]. The balance between coagulation and anticoagulation is necessary for uteroplacental circulation and organ perfusion in pregnant women. Enhanced thrombin generation in individuals with preeclampsia triggers fibrin formation and activates platelets and endothelial cells, resulting hypercoagulation [31]. Preeclamptic women exhibit abnormal coagulation profiles, with prolonged activated partial thromboplastin time (APTT), prothrombin time (PT), elevated D-dimer, and mean platelets volume [25]. However, natural anticoagulants like antithrombin III, protein C, and protein S decline. Jin et al. [25]. examined pregnant women's coagulation profiles during first trimester for later preeclampsia risk. Their results showed lower D-dimer, APTT, thrombin time, antithrombin, and fibrin degradation products in preeclampsia women at diagnosis compared to controls. Alterations in coagulation were not linked to severity or onset time. Early pregnancy coagulation profiling may predict preeclampsia development, enhancing preventive strategies for patient safety. Additionally, postpartum women are at high risk of simultaneous bleeding and thrombosis, requiring vigilance in management strategies. Tissue factor pathway inhibitor (TFPI) plays a significant role in modulating coagulation, fibrinolysis, and trophoblast invasion for successful pregnancies [46]. TFPI deficiency and altered fibrinolytic system are linked to defective spiral artery development [46,47]. Plasminogen, converted to plasmin, is a key enzyme in the fibrinolytic system which induces fibrin degradation to produce fibrin degradation products. The activating capacity of tissue-type plasminogen activator (t-PA) depends on fibrin presence. Preeclampsia is associated with defects in t-PA induced activation of plasminogen [47]. Endothelial dysfunction in preeclamptic women elevates t-PA and plasminogen activator inhibitor 1, signifying hypercoagulability. Godtfredsen et al. [47]. found elevated t-PA and plasminogen activator inhibitor 1 levels in preeclampsia, with lower plasminogen activator inhibitor 2 concentrations compared to controls. Plasminogen activator inhibitor 2 polymerize in both groups and no difference in fibrinolytic potential was observed. D-dimer increased and plasminogen/plasmin inhibitor and fibrin clot lysability time decreased in preeclampsia. They concluded that disruptions in the t-PA/plasminogen activator inhibitor 1 system, decreased plasminogen activator inhibitor 2, and increased fibrin lysability contribute to preeclampsia development. Altered concentrations in preeclamptic women suggest TFPI as potential predictive biomarkers for preeclampsia [46].

#### 6.7.4 HELLP syndrome in preeclampsia

HELLP syndrome is considered a severe complication of preeclampsia. HELLP syndrome involves microangiopathic hemolysis, liver enzyme elevation, and thrombocytopenia, often presenting with common symptoms like nausea, vomiting, headache, and visual changes. Thrombocytopenia arises from platelet consumption due to diffuse injury. Severe cases may progress to disseminated intravascular coagulation, characterized by decreased fibrinogen and antithrombin levels, and increased prothrombin time and fibrin [48].

#### 6.7.5 Role of microparticles

Under normal physiological conditions, MPs contribute to maintaining homeostasis. In comparison to normal pregnant women, preeclamptic women exhibit high levels of MPs derived from platelets, endothelial cells, syncytiotrophoblasts, leukocytes, and monocytes [49]. High levels of circulating membrane MPs exhibiting tissue factor and procoagulant phospholipids like phosphatidylserine trigger thrombin generation and are associated with vascular complications in pregnant women and the risk of fetal loss [50]. Maternal conditions associated with endothelial cell activation and immune system modulation may induce the release of MPs, leading to increased endothelial dysfunction, inflammation, and coagulation [49]. Circulating syncytiotrophoblast-derived MPs manifest placental injury [49]. Preeclampsia causes alterations in the circulating platelet MPs profile, which may affect hemostasis and increase the risk of preeclampsia-associated VTE [26]. Platelet MPs tend to activate endothelium, monocytes, and platelets and are involved in trophoblast dysfunction, abnormal placentation, imbalanced angiogenesis, and intravascular inflammation [49,51]. Elevated levels of circulating endothelial MPs in preeclamptic women indicate endothelial damage, and these MPs' count may serve as a biomarker for predicting severe endothelial dysfunction in pregnant women [49].

## 7 Prevention of preeclampsia and related complications

LDA is the recommended medication for preventing preeclampsia in high-risk women, using doses ranging from 50 to 150 mg daily, and starting between 12 and 28 weeks, continuing until delivery [52]. The most beneficial effects are seen when aspirin is started before 16 weeks’ gestation [1]. Aspirin is a non-steroidal anti-inflammatory drug that non-selectively and irreversibly inhibits the activity of cyclooxygenase, leading to a reduction of prostaglandins and TXA_2_. This effect results in an antithrombotic outcome by decreasing the TXA_2_/prostacyclin (PGI_2_) ratio [5]. The underlying mechanism of aspirin treatment to prevent preeclampsia is not clearly known. Proposed mechanisms include the inhibition of thromboxane production, enhancing the placentation process, inhibiting platelet aggregation to reduce placental infarction, and exerting anti-inflammatory effects while stabilizing endothelial function [4]. On the other hand, aspirin's efficacy in preventing preeclampsia varies, possibly due to factors beyond TXA_2_/PGI_2_ modulation, such as patient variability and gestational timing. Its greater effectiveness against early-onset preeclampsia suggests a role in placental development rather than maternal biochemical dysregulation [5]. Aspirin treatment inhibits platelets activation and inflammation, mitigating preeclampsia development [6]. LDA also improves trophoblast invasion and migration into the uterine arteries, disrupts cytokine production, and stimulates the production of the proangiogenic protein PlGF, thereby inhibiting apoptosis and premature remodeling of uterine arteries [28]. The first case report of aspirin’s potential use during pregnancy was reported in 1978 by Fitzgerald DJ [53], who described that in patients with preeclampsia thromboxane production was increased in conjunction with decreased prostaglandin; usually thromboxane is produced mainly in trophoblastic and stromal tissues, whereas PGI_2_ is produced mainly in vascular tissue. In preeclamptic pregnancy, the placenta produces seven times more thromboxane than prostacyclin. However, the prophylactic role of aspirin remains controversial due to varying results, with literature reporting a risk reduction ranging from 10 % to 68 %. This variability could be due to factors like aspirin dosage, timing of initiation, and risk assessment methods [5]. Despite aspirin's efficacy, a subgroup of women remains at high risk for preeclampsia regardless of treatment due to aspirin non-responsiveness. The underlying reason for this variability in responsiveness is unknown. Genetic differences, pharmacokinetics, and pharmacodynamics have been considered to play an essential role [7,8]. Given the large prevalence of preeclampsia, personalized management strategies are essential to optimize outcomes and mitigate drug side effects [7].

### 7.1 Aspirin treatment

#### 7.1.1 How and when

It is considered that initiation of aspirin treatment should be provided before 16 weeks of gestation, as placental development and remodeling of uterine spiral arteries are typically concluded by the 20th week of gestation [54]. Its effectiveness follows a dose-response pattern, with the greatest impact observed with 100 mg dose when taken before 16 weeks of gestation [5]. Aspirin was not effective when provided after the occurrence of preeclampsia due to placental factors such as shallow implantation [55]. Thus, administering aspirin before full placental formation is crucial to mitigate complications of pregnancy-induced hypertension [6]. The reason behind ineffectiveness of aspirin treatment initiation after 16 weeks of gestation is unknown and the effect of aspirin treatment on the molecular pathways involved in pathogenesis of preeclampsia is unclear.

The key characteristics of trials evaluating the use of low-dose aspirin for the prevention of preeclampsia are detailed in Table 1. Some studies have shown conflicting results, indicating no significant difference in reducing preeclampsia risk with aspirin. Meta-analyses suggest a dose-dependent effect, with doses ≥100 mg showing a stronger preventive impact—especially when started before 16 weeks—some individual trials yielded neutral results, likely due to heterogeneity in design, timing, and populations. The strongest evidence supports daily aspirin at 150 mg initiated before 16 weeks of gestation in high-risk women. This discrepancy may be attributed to diverse risk factors in preeclamptic women, compounded by various comorbidities and variations in aspirin dosage/timing. Earlier aspirin initiation may prevent preterm birth and small-for-gestational-age infants [6]. Richards et al.’s meta-analysis [6] found no preeclampsia risk reduction difference between early and late aspirin initiation. However, aspirin and/or dipyridamole significantly decreased preterm birth risk before 37 weeks, especially in hypertensive women. This suggests LDA could effectively prevent late preterm birth in hypertensive women. Moderate to late preterm birth is linked to placental vascular pathology, while very preterm birth is associated with infection and inflammation. Chronic hypertension notably predisposes pregnant women to vascular pathology compared to other preeclampsia risk factors. Aspirin’s efficacy in reducing late preterm birth in hypertensive women may stem from its action on placental vascular pathology. Further research is needed to comprehend aspirin's mechanism in preventing preterm birth.

**Table 1:** Selected landmark trials on low-dose aspirin use in preeclampsia prevention.

Authors, year	N. of patients	Inclusion criteria	Aspirin	Comparator arm	Onset (weeks)	Outcomes	Findings
Amro et al. (2025)[68]	343	BMI≥30 kg/m^2^ and at least 1 of 3 high-risk PE factors	162 mg	ASA 81 mg	12–20	PE with severe features	Among high-risk obese individuals, there was a 78 % probability of benefit that 162 mg aspirin compared with 81 mg will decrease the rate of preeclampsia with severe features
Ayala et al. (2013)[86]	350	History, risk factor	100 mg	Placebo	13	PE, preterm delivery, stillbirth, IUGR	Lower hazard ratio (HR) of serious adverse outcomes (0.35, 95 % [CI]: 0.22–0.56; *p* < 0.001); no increased risk of hemorrhage, with low-dose ASA relative to placebo (HR: 0.57, 95 % CI: 0.25–1.33; *p* = .194)
Caritis et al. (1998)[87]	523	High risk	60 mg	Placebo	<16	PE, preterm birth, SGA, abruptio placenta	Low-dose ASA did not reduce the incidence of PE significantly or improve perinatal outcomes in pregnant women at high risk for PE
CLASP (1994)[88]	9309	History, risk factors	60 mg	Placebo	12–32	PE, IUGR, stillbirth	Nor was there any significant effect on outcomes. ASA significantly reduce the likelihood of preterm delivery (19.7 % aspirin vs 22.2 % control; absolute reduction of 2.5 [SD 0.9] per 100 women treated; 2p = 0.003). ASA was not associated with a significant increase in placental hemorrhages
Goffinet (1996)[89]	970	History, risk factors	60 mg	Placebo	12–32	PE, preterm delivery, IUGR, birthweight, stillbirth	There were no significant differences between the treatment groups in terms of outcomes. ASA was not associated with a significant excess of maternal or fetal bleeding
Golding et al. (1998)[90]	6049	Nulliparity	60 mg	Placebo	12–32	Gestational HTN, PE, eclampsia, birthweight, preterm birth	No differences between patients in treatment with ASA and those on placebo in the development of hypertensive disorders OR was 1.02 [95 % CI 0.86–1.21]. They were, however, significantly more likely to suffer from bleeding disorders OR 1.40 (95 % CI 1.13–1.73)
Hermida et al. (1999) [91]	350	History, risk factors	100 mg	Placebo	12–16	IUGR, preterm birth, abruptio placenta	Effects of aspirin on blood pressure were significantly larger for women with a positive tolerance-hyperbaric test at the time of recruitment (*P*<.001)
Morris et al. (1996) [92]	102	Nulliparity and abnormal uteroplacental resistance at 18 weeks of gestation	100 mg	Placebo	17–19	Fetal growth restriction, PE, preterm birth, perinatal death	Prophylactic ASA therapy did not result in a significant reduction in pregnancy complications
Rolnik et al. (2017)[93]	1629	High risk for preterm PE	150 mg	Placebo	11–14	Preterm PE, PE, gestational HTN, SGA, stillbirth, abruption, preterm birth, poor fetal growth	In the ASA group, the incidence of preterm PE was reduced by 62 %
Villa et al. (2013)[94]	121	Abnormal uterine artery Doppler plus history	100 mg	Placebo	12–14	PE, gestational HTN, birthweight, SGA	Low-dose ASA did not reduce the rate of PE (relative risk [RR] 0.7, 95 % CI 0.3–1.7); gestational HTN (RR 1.6, 95 % CI 0.6–4.2)
Yu et al. (2003)[55]	560	High risk of uteroplacental insufficiency	150 mg	Placebo	22–24	Incidence of PE and the other consequences of impaired placentation	No significant differences between the ASA and placebo groups in either the incidence of PE (18 % vs. 19 %, *P* = .6)

ASA, acetylsalicylic acid; HNT, hypertension; IUGR, intrauterine growth restriction; PE, preeclampsia; SGA, small for gestational age.

The milestone study informing the guidelines is the large ASPRE trial (Aspirin versus Placebo in Pregnancies at High Risk for Preterm Preeclampsia) [56], that enrolled 1776 women at high risk for preeclampsia undergoing a combined multimarker screening (based on maternal characteristics, biophysical parameters, biochemical profile) and randomized patient to aspirin for preeclampsia prevention. Aspirin was administered at 150 mg at 11–14 weeks of gestation until 36 weeks of gestation. Preterm preeclampsia occurred in 13 participants (1.6 %) in the aspirin group, as compared with 35 (4.3 %) in the placebo group (odds ratio in the aspirin group, 0.38; 95 % confidence interval, 0.20 to 0.74; *P* = .004). Results were materially unchanged in a sensitivity analysis that took into account participants who had withdrawn or were lost to follow-up. Adherence was good, with a reported intake of 85 % or more of the required number of tablets in 79.9 % of the participants. There were no significant between-group differences in the incidence of neonatal adverse outcomes or other adverse events.

The Roberge’s metanalysis [57] analysed 45 randomized controlled trials that included a total of 20,909 pregnant women randomized to between 50–150 mg of aspirin daily. When aspirin was initiated at ≤16 weeks, there was a significant reduction and a dose-response effect for the prevention of preeclampsia (relative risk, 0.57; 95 % confidence interval, 0.43–0.75; *P* < .001; R^2^, 44 %; *P* = .036), severe preeclampsia (relative risk, 0.47; 95 % confidence interval, 0.26–0.83; *P* = .009; R^2^, 100 %; *P* = .008), and FGR (relative risk, 0.56; 95 % confidence interval, 0.44–0.70; *P* < .001; R^2^, 100 %; *P* = .044) with higher dosages of aspirin being associated with greater reduction of the 3 outcomes. When aspirin was initiated at >16 weeks, there was a smaller reduction of preeclampsia (relative risk, 0.81; 95 % confidence interval, 0.66–0.99; *P* = .04) without relationship with aspirin dosage (R^2^, 0 %; *P* = .941). Aspirin initiated at >16 weeks was not associated with a risk reduction or a dose-response effect for severe preeclampsia (relative risk, 0.85; 95 % confidence interval, 0.64–1.14; *P* = .28; R^2^, 0 %; *P* = .838) and FGR (relative risk, 0.95; 95 % confidence interval, 0.86–1.05; *P* = .34; R^2^, not available; *P* = .563).

Placental dysfunction accounts for 70 % of preterm preeclampsia, but it is associated with only 30 % of term preeclampsia. Thus, aspirin may better prevent preterm than term preeclampsia if this is the underlying mechanism, while limited evidence exists on aspirin's effectiveness against term preeclampsia. Currently, the aspirin dosages contrast among national guidelines worldwide, ranging from 60–162 mg/day. Therefore, further research is needed to find the optimal aspirin dosage [58]. The meta-analysis of Ghesquiere et al. [58]. found that starting aspirin at 150–162 mg/day between 11–14 weeks gestation reduced preterm preeclampsia compared to 75–81 mg/day. Higher doses also lowered the risk of severe preeclampsia. However, the aspirin dosage did not significantly reduce the risk of term or overall preeclampsia. The study suggests that 150–162 mg/day aspirin from 11–14 weeks may be preferable to the commonly used 75–81 mg/day to prevent preterm preeclampsia.

#### 7.1.2 Aspirin and hemorrhagic risk

The antiplatelet properties of aspirin are believed to prevent preeclampsia by decreasing local thrombosis in the maternal vessels that supply the placenta, thereby improving placental perfusion. However, the increased maternal blood flow to the placenta may result in higher hemorrhage risk during delivery, even after aspirin treatment cessation at 36 weeks of gestation. Aspirin treatment in second half of pregnancy can cause severe outcomes such as placental disruption [59,60]. Previous literature indicates that administering aspirin therapy to nulliparous women with a daily dose of 60 mg between 13 and 26 weeks of gestation is linked to a heightened risk of placental abruption. This might be attributed to the delayed initiation of aspirin treatment. By 16 to 18 weeks of gestation, placentation concludes, suggesting that delaying aspirin treatment in women with abnormal placentation may elevate the risk of placental abruption [4]. In contrast, previous literature has also reported that initiating aspirin treatment before 11 weeks of gestation in high-risk women did not decrease the risk of preeclampsia and gestational hypertension [61]. Inconsistencies among studies could be due to inaccurate stratification of risk factors and aspirin dosage. Future research should focus on the prophylactic effects of aspirin in different populations or populations at different risks [62]. Using an aspirin dosage higher than 150 mg significantly increases the risk of bleeding. However, several studies have reported that the risks of placental abruption, postpartum hemorrhage, cerebral hemorrhage, and perinatal mortality are not increased by LDA. Hastie et al. [59] found higher rates of intrapartum bleeding (2.9 % vs. 1.5 %) and postpartum hemorrhage (10.2 % vs. 7.8 %) in pregnant women using aspirin compared to non-users. They also noted increased risks of postpartum hematoma and neonatal intracranial hemorrhage, advising careful risk assessment before prescribing aspirin to pregnant women [59]. The insufficient accuracy of clinical tests predicting preeclampsia limits the risk stratification. Thus, it can sometimes be challenging to decide which women should be prescribed aspirin prophylactically [26]. Mendoza et al. [60]. explored the safety of discontinuing aspirin earlier. They found that stopping aspirin at 24 to 28 weeks of gestation was not inferior in preventing preterm preeclampsia in high-risk pregnant individuals with a normal sFlt-1:PlGF ratio. This suggests the potential for reducing the risk of peripartum bleeding. Further research is needed to understand the clinical implications of varying doses and treatment durations. The risks associated with aspirin affect both mothers and fetuses. Therefore, during pregnancy, monitoring for adverse effects such as gastrointestinal ulceration, hepatic and renal toxicity, bleeding, allergic reactions, and Rayleigh's syndrome is essential.

#### 7.1.3 Unmet needs

Practice guidelines on aspirin use in pregnancy showed heterogeneity in the indication for aspirin administration, optimal dosage, time to starting and ending therapy, safety and side effects. The starting time and dose of oral aspirin are controversial. A meta-analysis by Meher et al. [63]. showed that aspirin could reduce the risk of preeclampsia by 10 % and there was no difference in the effect of oral aspirin before and after 16 weeks of pregnancy on the risk of preeclampsia; instead a meta-analysis by Roberge et al. [57]. that included 45 randomized controlled trials found that aspirin starting at < 16 gestational weeks could effectively reduce the risk of preeclampsia (RR = 0.57), and the effect of daily oral aspirin > 100 mg was much more significant.

One of the unmet needs is whether the timing of aspirin administration may be more important than the dosage in achieving significant net benefits, with "timing" referring both to the time of day the medication is taken and the gestational age at which the therapy is initiated.

The ASPRE trial [56] was the only trial to use a previously developed, externally validated risk prediction model for predicting preeclampsia. The model was used to assess risk of preeclampsia leading to preterm birth in participants with singleton pregnancies at 11 to 12 weeks of gestation. The prediction algorithm estimated risk based on clinical history characteristics such as weight, height, maternal age, race/ethnicity, previous preeclampsia, family history of preeclampsia, chronic hypertension, diabetes type 1 or 2, systemic lupus erythematosus or antiphospholipid syndrome, and conception by *in vitro* fertilization. This was then supplemented with serum biomarker tests (PAPP-A and PlGF), mean arterial pressure and Doppler ultrasound of the uterine artery pulsatility index. It is not clear whether the greater effectiveness of aspirin seen in the ASPRE trial [56] relative to other large trials can be attributed to the risk assessment approach, and how much is due to other differences, such as the higher aspirin dosage, or the early initiation of aspirin at 12 to 13 weeks of gestation and continuation until 36 weeks or onset of labor. Whether the algorithm identifies a population particularly responsive to aspirin cannot be determined without further comparative research.

In a retrospective cohort study [64], patients who were at high risk for preeclampsia and were treated under a 162 mg prophylactic aspirin protocol had a significantly lower rate of preeclampsia than women who received the standard 81 mg dose regimen alongside no apparent increased risk for bleeding.

In a recent metanalysis [65] LDA (75–150 mg) significantly reduced preeclampsia incidence in high-risk women. Early initiation, particularly between 12 and 16 weeks, demonstrated enhanced efficacy. Higher doses, such as 150 mg, were associated with better outcomes compared to lower doses. Based on the analysis of 31 studies involving 28,318 pregnancies and 20 studies involving 26,551 pregnancies, the early initiation of aspirin significantly reduced the overall incidence of preeclampsia (RR = 0.63, CI: 0.47–0.84) and perinatal death risk (RR = 0.82, CI: 0.72–0.93), respectively [66].

Whether body weight may affect the efficacy of aspirin and deserves higher doses is also a matter of debate. Obesity may alter aspirin pharmacokinetics during pregnancy. The absorption of aspirin , especially enteric-coated aspirin, is reduced in obese pregnant patients [67]. In an open-label randomized trial (ASPREO) [68] among high-risk obese individuals, there was a 78 % probability of benefit that 162 mg aspirin compared with 81 mg will decrease the rate of preeclampsia with severe features. With the best estimate of a 12 % reduction when using 162 mg of aspirin compared with 81 mg of aspirin in this population.

A smaller study investigating aspirin use in pregnant women found that obesity was associated with lower concentration of aspirin metabolites at the 81 mg dose, but not at 162 mg. Interestingly, metabolite levels in obese pregnant women receiving 162 mg were comparable to those in non-obese women taking 81 mg [69]. These findings suggest that individuals with obesity may benefit from a higher aspirin dose [69]. In evaluating the optimal circadian timing of aspirin administration, a prospective randomized trial enrolled 350 pregnant individuals at elevated risk, assigning them to receive either 100 mg of aspirin or a placebo. The intervention began between 12 and 16 weeks of gestation and was administered daily—either upon waking, eight hours after waking, or at bedtime.

Findings from the study revealed that aspirin taken at bedtime was associated with a lower incidence of preeclampsia, gestational hypertension, preterm birth, and FGR, compared to aspirin taken earlier in the day.

Although the underlying mechanism is not fully understood, it is hypothesized that circadian variations in thromboxane and prostacyclin production may play a role, and that nighttime administration of aspirin may more effectively modulate biological regulators of blood pressure [70,71].

The long-term risks of low dose aspirin for the mother and her child are uncertain, as the included trials have not reported long-term outcomes. Several studies varied considerably about excluded populations and sample size. No studies reported head-to-head comparison of different protocols. Further research is needed to explore these grey areas and to address gaps in the research related to potential variability in the effectiveness of aspirin.

#### 7.1.4 Lower-than -expected aspirin responsiveness in preeclampsia

Aspirin demonstrates efficacy in 50 % of patients at risk for preeclampsia. The progression of preeclampsia, despite aspirin prophylaxis, has been attributed to aspirin non-responsiveness [72]. Aspirin resistance [72], defined as the inability of aspirin to reduce TXA_2_ production from platelets, leads to platelet activation and aggregation [73]. Aspirin non-responsiveness is multifactorial. In non-obstetric populations, factors such as platelet turnover, aspirin dosage, diabetes, smoking, and weight have been linked to aspirin non-responsiveness [8,74]. Incomplete adherence is the primary cause of aspirin low response, with a 46.3 % non-adherence rate reported in high-risk preeclampsia women. Aspirin reduces the risk of preeclampsia with ≥90 % adherence; but it does not lower the risk when adherence falls below 90 %. However, the high prevalence of inadequate aspirin adherence (<90 %) significantly correlates with increased incidences of preeclampsia, intrauterine growth restriction (IUGR), and preterm delivery [72,74]. The pharmacological effects of aspirin interact with genetic polymorphisms in various genes, including those encoding glycoproteins, cyclooxygenases, adenosine diphosphate receptors, and hemostasis proteins, observed in CVD. Investigating pharmacogenetic effects of aspirin in preeclamptic women may elucidate mechanisms of aspirin non-responsiveness in those at high risk of developing preeclampsia despite therapy [7]. Aspirin undergoes polymorphic metabolism, with common genetic variants found in different racial and ethnic groups. These variants, along with their mixtures, may contribute to the considerable variability in both adverse reactions and efficacy of aspirin [73].

Obesity is recognized as an important risk factor for preeclampsia, and evidence suggests that LDA may be less effective in this population [67]. There are several potential mechanisms to explain this phenomenon, including increased platelet reactivity, increased platelet turnover, reduced bioavailability of LDA, as well as higher levels of oxidative stress and inflammation in obese individuals [67]. Given the increased rates of aspirin low responsiveness in obese individuals, the traditional low dose aspirin of 60 and 81 mg available in the United States may be insufficient to fully suppress TXA_2_ production in this population. Exploring the pharmacokinetics and pharmacodynamics of aspirin during pregnancy would be beneficial for determining the optimal safe and effective dose of aspirin in high-risk pregnancies [8].

### 7.2 Low‐molecular‐weight heparin

In the treatment of VTE during pregnancy, LMWH is preferred as it does not cross the placenta, ensuring fetal safety [75]. Literature suggests higher risks with heparin and its derivatives in preeclamptic patients compared to LDA, including bleeding and heparin-induced thrombocytopenia. However, it could be the limitation of large trials that may underweight LMWH efficacy by including all preeclamptic patients without considering underlying risk factors [76]. Chen et al. [77]. conducted a systematic review and meta-analysis indicating that the combination of LMWH and LDA is effective for the prevention preeclampsia, preterm birth, and FGR in high-risk women without thrombophilia compared to LDA alone. LMWH alone did not show prophylactic efficacy against preeclampsia when aspirin was not used concurrently. This may be because pregnant women requiring LDA are predisposed to thrombotic events and placental complications like preeclampsia. Additionally, LMWH may enhance aspirin's effect. The reduction in preterm birth and FGR may signify LMWH's role in preventing preeclampsia or targeting its pathogenesis. Notably, it has been reported in the literature that unfractionated heparin did not cause a significant reduction in placental thrombotic lesions in high-risk women [78]. These findings suggest that LMWH reduce the complications of preeclampsia through non‐anticoagulant pathways, as both unfractionated and LMWH exhibit various non‐anticoagulant function along with antithrombotic functions [76].

### 7.3 Statins

Statins, inhibitors of HMG-CoA reductase, are effective in primary and secondary prevention of cardiovascular mortality and morbidity. Statins are promising candidates for the prevention and treatment of preeclampsia due to their multiple and pleiotropic mechanisms of action [79]. They upregulate endothelial NO synthase, promoting NO production, and enhance the release of VEGF and PlGF [80]. Statins also reduce sFlt-1 and sEng levels, while upregulating heme oxygenase-1 (HO-1) expression in endothelial and smooth muscle cells. Some studies suggest that statins activate the HO-1/CO pathway, suppressing sFlt-1 production [80]. Additionally, statins possess anti-inflammatory properties, lowering hs-C reactive protein even in patients with normal cholesterol levels [81]. They promote Th2 anti-inflammatory cytokines and reduce Th1 pro-inflammatory cytokines [82]. These effects, combined with actions on free radicals and smooth muscle cell proliferation, make statins strong candidates for preeclampsia management.

Prior findings raised concerns about statins' teratogenicity. However, research suggests that pravastatin's hydrophilicity prevents fetal malformation risk by restricting placental crossing, indicating its potential safety in pregnancy [83]. Currently, its role in preventing preeclampsia is under study to counter the angiogenic imbalance, a key feature of preeclampsia, and to restore endothelial dysfunction. Toghi et al. [84]. proposed pravastatin as a promising treatment for preeclampsia, demonstrating its ability to lower systolic blood pressure and prevent placental weight loss in a mouse model. Its antioxidant properties protect against oxidative stress-induced metalloproteinase-2 activation and promote NO-dependent vasodilation from endothelium. Fruci et al. [85]. demonstrated that administering a daily 40 mg dose of pravastatin reduced the severity of liver, kidney, and hematological complications, as indicated by lower transaminase and creatinine levels, yet higher platelet counts. Furthermore, pravastatin did not reduce placental lesions in women with preeclampsia and IUGR, suggesting late second-trimester administration could not reverse established placental dysfunction. Pravastatin may stabilize the disease by enhancing prenatal weight gain and prolonging pregnancy duration, potentially mitigating maternal disease severity. Santoyo et al. [49]. studied pravastatin's effects (20 mg/day) from 35 to 37 weeks of gestation until delivery on circulating extracellular vesicles (EVs) in high-risk women for term preeclampsia. They observed a significant reduction in plasma concentration of large EVs from platelets (42 %), leukocytes (25 %), monocytes (61 %), endothelial cells (69 %), activated endothelial cells (55 %), and syncytiotrophoblast cells (44 %), suggesting that statins may be beneficial in reducing endothelial dysfunction and pro-inflammatory and pro-coagulatory conditions associated with preeclampsia.

Given the promising preliminary findings and strong biological rationale, large-scale randomized controlled trials are urgently needed to evaluate the efficacy and safety of statins as a preventive strategy against preeclampsia.

## 8 Limitations

There are a number of limitations to this review. Firstly, literature selection, while purposeful, is susceptible to subjective judgment and, in its narrative form, precludes systematic quantification of study quality or risk of bias. Secondly, indirect comparability and meta-analytic integration are compromised due to high heterogeneity in the included studies’ design, populations, endpoints, and interventions. Third, some of the results will be subject to revision as new information becomes available due to the dynamic nature of the evidence, especially the issue of new therapies and biomarkers. In spite of these challenges, we are convinced that this review represents an informed integration of major clinical and mechanistic results.

## 9 Conclusions

Preeclampsia represents a major maternal health challenge, with complex and multifactorial pathogenesis, centered on placental dysfunction, endothelial injury, and thrombo-inflammatory pathways. While low-dose aspirin remains the most established preventive strategy, questions persist regarding optimal use and individual response. Future research should prioritize personalized prevention strategies and the development of reliable biomarkers to improve maternal and fetal outcomes.

This review was supported by a grant from “NextGenerationEU” – MUR Promotion and Development Fund- DM 737/2021, “The AWARENESS Study”- vAriability in loW-dose Aspirin REspoNse in prEgnant women at high riSk for preeclampSia: implications for obstetric outcome, maternal and fetal cardiovascular function

## 10 Disclosures

FS: Advisory Board Bayer. GR: Research grant/contract: Bayer, BMS- Janssen; speaker fee: Bayer, Boehringer Ingelheim. The other authors have nothing to disclose.

## Author declaration

This review was supported by a grant from “NextGenerationEU” – MUR Promotion and Development Fund- DM 737/2021, “The AWARENESS Study”- vAriability in loW-dose Aspirin REspoNse in prEgnant women at high riSk for preeclampSia: implications for obstetric outcome, maternal and fetal cardiovascular function.

We confirm that the manuscript has been read and approved by all named authors and that there are no other persons who satisfied the criteria for authorship but are not listed. We further confirm that the order of authors listed in the manuscript has been approved by all of us.

We confirm that we have given due consideration to the protection of intellectual property associated with this work and that there are no impediments to publication, including the timing of publication, with respect to intellectual property. In so doing we confirm that we have followed the regulations of our institutions concerning intellectual property.

We understand that the Corresponding Author is the sole contact for the Editorial process (including Editorial Manager and direct communications with the office). He/she is responsible for communicating with the other authors about progress, submissions of revisions and final approval of proofs. We confirm that we have provided a current, correct email address which is accessible by the Corresponding Author.

## CRediT authorship contribution statement

**Chiara Martini:** Validation, Writing – original draft. **Zeeba Saeed:** Validation, Writing – original draft. **Paola Simeone:** Validation, Writing – original draft. **Stefano Palma:** Validation, Visualization. **Mirella Ricci:** Validation, Visualization. **Allegra Arata:** Validation, Visualization. **Anna Sorella:** Validation, Visualization. **Rossella Liani:** Validation, Visualization. **Fabrizio Ricci:** Supervision, Validation. **Francesco D’Antonio:** Supervision, Validation. **Anna Vittoria Mattioli:** Supervision, Validation. **Sabina Gallina:** Supervision, Validation. **Francesca Santilli:** Validation, Funding acquisition, Supervision, Writing – review & editing. **Giulia Renda:** Supervision, Writing – review & editing, Conceptualization, Methodology, Validation.

## Declaration of competing interest

The authors declare the following financial interests/personal relationships which may be considered as potential competing interests:

Francesca Santilli reports financial support was provided by European Union. Francesca Santilli reports a relationship with Bayer AG that includes: board membership. Giulia Renda reports a relationship with Bayer AG that includes: funding grants and speaking and lecture fees. Giulia Renda reports a relationship with Janssen Pharmaceuticals Inc that includes: funding grants. Giulia Renda reports a relationship with Boehringer Ingelheim GmbH that includes: speaking and lecture fees. If there are other authors, they declare that they have no known competing financial interests or personal relationships that could have appeared to influence the work reported in this paper.

## References

[1] (2020). Gestational hypertension and preeclampsia. Obstet Gynecol.

[2] Dimitriadis E., Rolnik D.L., Zhou W., Estrada-Gutierrez G., Koga K., Francisco R.P .V.. (2023). Author correction: pre-eclampsia. Nat Rev Dis Primers.

[3] Poon L.C., Shennan A., Hyett J.A., Kapur A., Hadar E., Divakar H. (2019). The International Federation of Gynecology and Obstetrics (FIGO) initiative on pre-eclampsia: a pragmatic guide for first-trimester screening and prevention. Int J Gynecol Obstet.

[4] Rolnik D.L., Nicolaides K.H., Poon L.C. (2022). Prevention of preeclampsia with aspirin. Am J Obs Gynecol.

[5] Shanmugalingam R., Hennessy A., Makris A. (2019). Aspirin in the prevention of preeclampsia: the conundrum of how, who and when. J Hum Hypertens.

[6] Richards E.M.F., Giorgione V., Stevens O., Thilaganathan B. (2023). Low-dose aspirin for the prevention of superimposed preeclampsia in women with chronic hypertension: a systematic review and meta-analysis. Am J Obs Gynecol.

[7] Chaemsaithong P., Biswas M., Lertrut W., Warintaksa P., Wataganara T., Poon L.C.Y. (2024). Pharmacogenomics of Preeclampsia therapies: current evidence and future challenges for clinical implementation. Best Pr Res Clin Obs Gynaecol.

[8] Navaratnam K., Alfirevic A., Jorgensen A., Alfirevic Z. (2018). Aspirin non-responsiveness in pregnant women at high-risk of pre-eclampsia. Eur J Obstet Gynecol Reprod Biol.

[9] Rana S., Lemoine E., Granger J.P., Karumanchi S.A. (2019). Preeclampsia. Circ Res.

[10] (2020). Correction to: preeclampsia: pathophysiology, challenges, and perspectives. Circ Res.

[11] Tomimatsu T., Mimura K., Matsuzaki S., Endo M., Kumasawa K., Preeclampsia Kimura T. (2019). Maternal systemic vascular disorder caused by generalized endothelial dysfunction due to placental antiangiogenic factors. Int J Mol Sci.

[12] Tyrmi J.S., Kaartokallio T., Lokki A.I., Jääskeläinen T., Kortelainen E., Ruotsalainen S. (2023). Genetic risk factors associated with preeclampsia and hypertensive disorders of pregnancy. JAMA Cardiol.

[13] Piechota W., Staszewski A. (1992). Reference ranges of lipids and apolipoproteins in pregnancy. Eur J Obs Gynecol Reprod Biol.

[14] Brizzi P., Tonolo G., Esposito F., Puddu L., Dessole S., Maioli M. (1999). Lipoprotein metabolism during normal pregnancy. Am J Obs Gynecol.

[15] Vrijkotte T.G.M., Krukziener N., Hutten B.A., Vollebregt K.C., van Eijsden M., Twickler M.B. (2012). Maternal lipid profile during early pregnancy and pregnancy complications and outcomes: the ABCD study. J Clin Endocrinol Metab.

[16] Belo L., Caslake M., Gaffney D., Santos-Silva A., Pereira-Leite L., Quintanilha A. (2002). Changes in LDL size and HDL concentration in normal and preeclamptic pregnancies. Atherosclerosis.

[17] Ouidir M., Zeng X., Workalemahu T., Shrestha D., Grantz K.L., Mendola P. (2020). Early pregnancy dyslipidemia is associated with placental DNA methylation at loci relevant for cardiometabolic diseases. Epigenomics.

[18] Mszar R., Gopal D.J., Chowdary R., Smith C.L., Dolin C.D., Irwin M.L. (2021). Racial/ethnic disparities in screening for and awareness of high cholesterol among pregnant women receiving prenatal care. J Am Heart Assoc.

[19] Poornima I.G., Indaram M., Ross J.D., Agarwala A., Wild R.A. (2022). Hyperlipidemia and risk for preclampsia. J Clin Lipidol.

[20] Fogacci S., Fogacci F., Banach M., Michos E.D., Hernandez A.V, Lip G.Y.H. (2020). Vitamin D supplementation and incident preeclampsia: a systematic review and meta-analysis of randomized clinical trials. Clin Nutr.

[21] Meertens L.J.E., Scheepers H.C.J., Willemse J.P.M.M., Spaanderman M.E.A., Smits L.J.M. (2018). Should women be advised to use calcium supplements during pregnancy? A decision analysis. Matern Child Nutr.

[22] Vanderlelie J., Scott R., Shibl R., Lewkowicz J., Perkins A., Scuffham P.A. (2016). First trimester multivitamin/mineral use is associated with reduced risk of pre-eclampsia among overweight and obese women. Matern Child Nutr.

[23] Redman C.W., Sargent I.L. (2005). Latest advances in understanding preeclampsia. Sci (1979).

[24] Phipps E., Prasanna D., Brima W., Preeclampsia Jim B. (2016). Updates in pathogenesis, definitions, and guidelines. Clin J Am Soc Nephrol.

[25] Jin P.P., Ding N., Dai J., Liu X.Y., Mao P.M. (2023). Investigation of the relationship between changes in maternal coagulation profile in the first trimester and the risk of developing preeclampsia. Heliyon.

[26] Raia-Barjat T., Edebiri O., Ni Ainle F. (2022). Preeclampsia and venous thromboembolism: pathophysiology and potential therapy. Front Cardiovasc Med.

[27] Guerby P., Tasta O., Swiader A., Pont F., Bujold E., Parant O. (2021). Role of oxidative stress in the dysfunction of the placental endothelial nitric oxide synthase in preeclampsia. Redox Biol.

[28] Dutta Kumar, Hyett Salomon (2019). Molecular targets of aspirin and prevention of preeclampsia and their potential association with circulating extracellular vesicles during pregnancy. Int J Mol Sci.

[29] Umezuluike B.S., Anikwe C.C., Nnachi O.C., Iwe B.C.A., Ifemelumma C.C., Dimejesi I.B.O. (2021). Correlation of platelet parameters with adverse maternal and neonatal outcomes in severe preeclampsia: a case-control study. Heliyon.

[30] Nowaczyk J., Poniedziałek B., Rzymski P., Sikora D., Ropacka-Lesiak M. (2022). Platelets in fetal growth restriction: role of reactive oxygen species, oxygen metabolism, and aggregation. Cells.

[31] Han C., Huang P., Lyu M., Dong J. (2020). Oxidative stress and preeclampsia-associated prothrombotic State. Antioxidants.

[32] Bokslag A., van Weissenbruch M., Mol B.W., de Groot C.J.M. (2016). Preeclampsia; short and long-term consequences for mother and neonate. Early Hum Dev.

[33] Irani R.A., Xia Y. (2008). The functional role of the renin–Angiotensin system in pregnancy and preeclampsia. Placenta.

[34] Wallukat G., Homuth V., Fischer T., Lindschau C., Horstkamp B., Jüpner A. (1999). Patients with preeclampsia develop agonistic autoantibodies against the angiotensin AT1 receptor. J Clin Investig.

[35] Sircar M., Thadhani R., Karumanchi S.A. (2015). Pathogenesis of preeclampsia. Curr Opin Nephrol Hypertens.

[36] Moghaddas Sani H, Zununi Vahed S, Ardalan M. (2019). Preeclampsia: a close look at renal dysfunction. Biomed Pharmacother.

[37] Pankiewicz K., Szczerba E., Maciejewski T., Fijałkowska A. (2019). Non-obstetric complications in preeclampsia. Menopausal Rev.

[38] Miller E.C., Vollbracht S. (2021). Neurology of Preeclampsia and Related disorders: an update in Neuro-obstetrics. Curr Pain Headache Rep.

[39] Wagner S.J., Acquah L.A., Lindell E.P., Craici I.M., Wingo M.T., Rose C.H. (2011). Posterior reversible encephalopathy syndrome and eclampsia: pressing the case for more aggressive blood pressure control. Mayo Clin Proc.

[40] Pfeffer T.J., Hilfiker-Kleiner D. (2018). Pregnancy and heart disease: pregnancy-associated hypertension and peripartum cardiomyopathy. Curr Probl Cardiol.

[41] Tassi A., Sala A., Mazzera I., Restaino S., Vizzielli G., Driul L. (2023). Long-term outcomes of patients with preeclampsia, a review of the literature. Hypertens Pregnancy.

[42] Hong K., Kim S.H., Cha D.H., Park H.J. (2021). Defective uteroplacental vascular remodeling in preeclampsia: key molecular factors leading to long term cardiovascular disease. Int J Mol Sci.

[43] Stanhewicz A.E., Dillon G.A., Serviente C., Alexander L.M. (2022). Acute systemic inhibition of inflammation augments endothelium-dependent dilation in women with a history of preeclamptic pregnancy. Pregnancy Hypertens.

[44] Sciscione A. (2003). Acute pulmonary edema in pregnancy. Obstet Gynecol.

[45] Park J.E., Park Y., Yuk J.S. (2021). Incidence of and risk factors for thromboembolism during pregnancy and postpartum: a 10-year nationwide population-based study. Taiwan J Obs Gynecol.

[46] Kobayashi H., Matsubara S., Yoshimoto C., Shigetomi H., Imanaka S. (2023). Tissue factor pathway inhibitors as potential targets for understanding the pathophysiology of preeclampsia. Biomedicines.

[47] Godtfredsen A.C., Sidelmann J.J., Dolleris B.B., Jørgensen J.S., Johansen E.K.J., Pedersen M.F.B. (2022). Fibrinolytic changes in women with preeclampsia. Clin Appl Thromb/Hemost.

[48] Haram K., Svendsen E., Abildgaard U. (2009). The HELLP syndrome: clinical issues and management. A review. BMC Pregnancy Childbirth.

[49] Santoyo J.M., Noguera J.A., Avilés F., Hernández-Caselles T., de Paco-Matallana C., Delgado J.L. (2023). Pravastatin reduces plasma levels of extracellular vesicles in pregnancies at high risk of term preeclampsia. Front Pharmacol.

[50] Van Dreden P., Lefkou E., Ka A., Sfakianoudis K., Rousseau A., Grusse M. (2022). Endothelial cell activation and thrombin generation assessment for the risk of severe early onset preeclampsia. The ROADMAP-EOP Study. Clin Appl Thromb/Hemost.

[51] Dionisio L.M., Favero G.M. (2023). Platelet indices and angiogenesis markers in hypertensive disorders of pregnancy. Int J Lab Hematol.

[52] Di Girolamo R., Alameddine S., Khalil A., Santilli F., Rizzo G., Maruotti G.M. (2023). Clinical practice guidelines on the use of aspirin in pregnancy: systematic review. Eur J Obs Gynecol Reprod Biol.

[53] Fitzgerald D.J., Mayo G., Catella F., Entman S.S., FitzGerald G.A. (1987). Increased thromboxane biosynthesis in normal pregnancy is mainly derived from platelets. Am J Obs Gynecol.

[54] Bujold E., Roberge S., Lacasse Y., Bureau M., Audibert F., Marcoux S. (2010). Prevention of preeclampsia and intrauterine growth restriction with aspirin started in early pregnancy. Obstet Gynecol.

[55] Yu C.K.H., Papageorghiou A.T., Parra M., Palma Dias R., Nicolaides K.H., Fetal Medicine Foundation Second Trimester Screening Group (2003). Randomized controlled trial using low-dose aspirin in the prevention of pre-eclampsia in women with abnormal uterine artery Doppler at 23 weeks’ gestation. Ultrasound Obs Gynecol.

[56] Rolnik D.L., Wright D., Poon L.C., O’Gorman N., Syngelaki A., de Paco Matallana C. (2017). Aspirin versus Placebo in pregnancies at high risk for preterm preeclampsia. N Engl J Med.

[57] Roberge S., Nicolaides K., Demers S., Hyett J., Chaillet N., Bujold E. (2017). The role of aspirin dose on the prevention of preeclampsia and fetal growth restriction: systematic review and meta-analysis. Am J Obs Gynecol.

[58] Ghesquiere L., Guerby P., Marchant I., Kumar N., Zare M., Foisy M.A. (2023). Comparing aspirin 75 to 81 mg vs 150 to 162 mg for prevention of preterm preeclampsia: systematic review and meta-analysis. Am J Obs Gynecol MFM.

[59] Hastie R., Tong S., Wikström A.K., Sandström A., Hesselman S., Bergman L. (2021). Aspirin use during pregnancy and the risk of bleeding complications: a Swedish population-based cohort study. Am J Obs Gynecol.

[60] Mendoza M., Bonacina E., Garcia-Manau P., López M., Caamiña S., Vives À. (2023). Aspirin discontinuation at 24 to 28 weeks’ Gestation in pregnancies at high risk of preterm preeclampsia. JAMA.

[61] Chaemsaithong P., Cuenca-Gomez D., Plana M.N., Gil M.M., Poon L.C. (2020). Does low-dose aspirin initiated before 11 weeks’ gestation reduce the rate of preeclampsia?. Am J Obs Gynecol.

[62] hui Liu Y, shen Zhang Y, yi Chen J, jian Wang Z, xin Liu Y, qi Li J (2023). Comparative effectiveness of prophylactic strategies for preeclampsia: a network meta-analysis of randomized controlled trials. Am J Obs Gynecol.

[63] Meher S., Duley L., Hunter K., Askie L. (2017). Antiplatelet therapy before or after 16 weeks’ gestation for preventing preeclampsia: an individual participant data meta-analysis. Am J Obs Gynecol.

[64] Ayyash M., Goyert G., Garcia R., Khangura R., Pitts D., Jacobsen G. (2024). Efficacy and safety of Aspirin 162 mg for preeclampsia prophylaxis in high-risk patients. Am J Perinatol.

[65] Saxena U., Lachyan A., Debnath A., Gupta S., Yadav A., Kishore J. Effectiveness of low-dose aspirin (75-150 mg) in preventing preeclampsia among high-risk pregnant women: a systematic review and meta-analysis of randomized controlled trials.

[66] Komoróczy B., Váncsa S., Váradi A., Hegyi P., Vágási V., Baradács I. (2025). Optimal aspirin dosage for the prevention of preeclampsia and other adverse pregnancy outcomes: a systematic review and meta-analysis of randomized controlled trials. J Clin Med.

[67] Norgard N.B. (2018). Obesity and altered aspirin pharmacology. Clin Pharmacokinet.

[68] Amro F.H., Blackwell S.C., Pedroza C., Backley S., Bitar G., Daye N. (2025). Aspirin 162 mg vs 81 mg for preeclampsia prophylaxis in high-risk obese individuals: a comparative effectiveness open-label randomized trial (ASPREO). Am J Obs Gynecol.

[69] Rood K.M., Ma’ayeh M., Abdelwahab M., Paglione M., Abbott N., Hill K. (2023). The effect of maternal body mass index on optimal dosing of aspirin for preeclampsia prevention. Am J Obs Gynecol.

[70] Ayala D.E., Ucieda R., Hermida R.C. (2013). Chronotherapy with low-dose aspirin for prevention of complications in pregnancy. Chronobiol Int.

[71] Snoep J.D., Hovens M.M.C., Pasha S.M., Frölich M., Pijl H., Tamsma J.T. (2009). Time-dependent effects of low-dose aspirin on plasma renin activity, aldosterone, cortisol, and catecholamines. Hypertension.

[72] Stubert J., Hinz B., Berger R. (2023). The role of acetylsalicylic acid in the prevention of pre-eclampsia, fetal growth restriction, and preterm birth. Dtsch Arztebl Int.

[73] Tolcher M.C., Sangi-Haghpeykar H., Mendez-Figueroa H., Aagaard K.M. (2020). Low-dose aspirin for preeclampsia prevention: efficacy by ethnicity and race. Am J Obs Gynecol MFM.

[74] Shanmugalingam R., Wang X., Motum P., Fulcher I., Lee G., Kumar R. (2020). Clinical influence of nonadherence with prophylactic aspirin in preventing preeclampsia in high-risk pregnancies. Hypertension.

[75] Mansory E.M., Alphonsus L., Hutson J.R., de Vrijer B., Lazo-Langner A. (2023). Anticoagulant prophylaxis in pregnant women with a history of venous thromboembolism: a systematic review and meta-analysis. Thromb Update.

[76] Wat J.M., Audette M.C., Kingdom J.C. (2018). Molecular actions of heparin and their implications in preventing pre-eclampsia. J Thromb Haemost.

[77] Chen J., Huai J., Yang H. (2024). Low-molecular-weight heparin for the prevention of preeclampsia in high-risk pregnancies without thrombophilia: a systematic review and meta-analysis. BMC Pregnancy Childbirth.

[78] D’Souza R., Keating S., Walker M., Drewlo S., Kingdom J. (2014). Unfractionated heparin and placental pathology in high-risk pregnancies: secondary analysis of a pilot randomized controlled trial. Placenta.

[79] Bronson F.H. (1988). Effect of food manipulation on the GnRH-LH-estradiol axis of young female rats. Am J Physiol.

[80] Wolfrum S., Jensen K.S., Liao J.K. (2003). Endothelium-dependent effects of statins. Arter Thromb Vasc Biol.

[81] Omori H., Nagashima H., Tsurumi Y., Takagi A., Ishizuka N., Hagiwara N. (2002). Direct in vivo evidence of a vascular statin: a single dose of cerivastatin rapidly increases vascular endothelial responsiveness in healthy normocholesterolaemic subjects. Br J Clin Pharmacol.

[82] Greenwood J., Steinman L., Zamvil S.S. (2006). Statin therapy and autoimmune disease: from protein prenylation to immunomodulation. Nat Rev Immunol.

[83] Costantine M.M., Cleary K., Hebert M.F., Ahmed M.S., Brown L.M., Ren Z. (2016). Safety and pharmacokinetics of pravastatin used for the prevention of preeclampsia in high-risk pregnant women: a pilot randomized controlled trial. Am J Obs Gynecol.

[84] Toghi C.J., Martins L.Z., Pacheco L.L., Caetano E.S.P., Mattos B.R., Rizzi E. (2023). Pravastatin prevents increases in activity of metalloproteinase-2 and oxidative stress, and enhances endothelium-derived nitric oxide-dependent vasodilation in gestational hypertension. Antioxidants.

[85] Fruci S., Salvi S., Moresi S., Gallini F., Dell’Aquila M., Arena V. (2023). Pravastatin for severe preeclampsia with growth restriction: placental findings and infant follow-up. Eur J Obstet Gynecol Reprod Biol.

[86] Ayala D.E., Ucieda R., Hermida R.C. (2013). Chronotherapy with low-dose aspirin for prevention of complications in pregnancy. Chronobiol Int.

[87] Caritis S., Sibai B., Hauth J., Lindheimer M.D., Klebanoff M., Thom E. (1998). Low-dose aspirin to prevent preeclampsia in women at high risk. N Engl J Med.

[88] (1994). CLASP: a randomised trial of low-dose aspirin for the prevention and treatment of pre-eclampsia among 9364 pregnant women. CLASP (Collaborative Low-dose Aspirin Study in Pregnancy) Collaborative Group. Lancet.

[89] (1996). ECPPA: randomised trial of low dose aspirin for the prevention of maternal and fetal complications in high risk pregnant women. BJOG.

[90] Golding J. (1998). A randomised trial of low dose aspirin for primiparae in pregnancy. BJOG.

[91] Hermida R.C., Ayala D.E., Fernández J.R., Mojón A., Alonso I., Silva I. (1999). Administration time–Dependent effects of Aspirin in women at differing risk for preeclampsia. Hypertension.

[92] MORRIS J., FAY R., ELLWOOD D., COOK C., DEVONALD K. (1996). A randomized controlled trial of Aspirin in patients with abnormal uterine artery blood flow. Obstet Gynecol.

[93] Rolnik D.L., Wright D., Poon L.C.Y., Syngelaki A., O’Gorman N., de Paco Matallana C. (2017). ASPRE trial: performance of screening for preterm pre-eclampsia. Ultrasound Obstet Gynecol.

[94] Villa P., Kajantie E., Räikkönen K., Pesonen A., Hämäläinen E., Vainio M. (2013). Aspirin in the prevention of pre-eclampsia in high-risk women: a randomised placebo-controlled PREDO Trial and a meta-analysis of randomised trials. BJOG.

